# Droplet Digital PCR versus qPCR for gene expression analysis with low abundant targets: from variable nonsense to publication quality data

**DOI:** 10.1038/s41598-017-02217-x

**Published:** 2017-05-25

**Authors:** Sean C. Taylor, Genevieve Laperriere, Hugo Germain

**Affiliations:** 1 Bio-Rad Laboratories, Inc., Hercules, CA 94547 USA; 2 Department of Chemistry, Biochemistry and Physics, Université du Québec à Trois-Rivières, 3351 boul. des Forges, Trois-Rivières, QC G9A 5H7 Canada

## Abstract

Quantitative PCR (qPCR) has become the gold standard technique to measure cDNA and gDNA levels but the resulting data can be highly variable, artifactual and non-reproducible without appropriate verification and validation of both samples and primers. The root cause of poor quality data is typically associated with inadequate dilution of residual protein and chemical contaminants that variably inhibit Taq polymerase and primer annealing. The most susceptible, frustrating and often most interesting samples are those containing low abundant targets with small expression differences of 2-fold or lower. Here, Droplet Digital PCR (ddPCR) and qPCR platforms were directly compared for gene expression analysis using low amounts of purified, synthetic DNA in well characterized samples under identical reaction conditions. We conclude that for sample/target combinations with low levels of nucleic acids (Cq ≥ 29) and/or variable amounts of chemical and protein contaminants, ddPCR technology will produce more precise, reproducible and statistically significant results required for publication quality data. A stepwise methodology is also described to choose between these complimentary technologies to obtain the best results for any experiment.

## Introduction

Data from qPCR experiments are taken within each enzymatic reaction curve at the quantification cycle (Cq). Therefore, optimization is critical for each primer pair such that reaction efficiency is consistent between all samples and acceptable (between 90% to 110%) with sample contaminants diluted adequately to assure that all reactions and associated Cq values are within the efficient range of the respective standard curves^1^. Poorly optimized reactions can result in artifactual Cq values and misinterpreted data that are difficult or even impossible to reproduce^2, 3^. For absolute quantification, data analysis is further complicated by the different sources of DNA from which the samples and standard curves are derived with unique backgrounds and contaminants that can variably affect the activity of Taq polymerase giving misleading results^4^. The Minimum Information for the Publication of Quantitative Real-Time PCR Experiments (MIQE) guidelines and related articles published thereafter define a rigorous methodology for designing qPCR experiments that assures publication of reproducible and high quality data^5–7^. The consequence of ignoring MIQE-guided protocols has led to the retraction of multiple articles over the past several years and remains a major frustration in the scientific community^8^.

Droplet Digital PCR (ddPCR) is a recent technology that has become commercially available since 2011^9, 10^. As with qPCR, ddPCR technology utilizes Taq polymerase in a standard PCR reaction to amplify a target DNA fragment from a complex sample using pre-validated primer or primer/probe assays. However, there are two distinct differences: 1) the partitioning of the PCR reaction into thousands of individual reaction vessels prior to amplification and 2) the acquisition of data at reaction end point. These factors offer the advantage of direct and independent quantification of DNA without standard curves giving more precise and reproducible data versus qPCR especially in the presence of sample contaminants that can partially inhibit Taq polymerase and/or primer annealing^11–13^. In addition, end-point measurement enables nucleic acid quantitation independently of the reaction efficiency, resulting in a positive-negative call for every droplet and greater amenability to multiplexed detection of target molecules^14^. Thereby, ddPCR technology can be used for extremely low-target quantitation from variably contaminated samples where the sample dilution requirements to assure consistent and acceptable reaction efficiency, primer annealing and Cq values for qPCR would likely lead to undetectable target levels^11, 15^.

In this study, synthetic DNA samples were used to directly compare and contrast qPCR with ddPCR technologies under common experimental conditions that can generate variable results in typical gene expression studies. In samples with low concentrations of nucleic acids and variable amounts of Taq inhibitors, ddPCR technology was shown to convert uninterpretable results generated from qPCR to highly quantitative and reproducible data.

## Results

### Experimental design to assess data quality between the qPCR and ddPCR acquisition platforms

Since the goal of the study was to directly compare the data quality between the qPCR and ddPCR platforms, care was taken to assure that the experimental design minimized all differences with the exception of the data acquisition platform (ie: qPCR versus ddPCR technology). A single reaction mix was therefore produced for each sample and for all experiments which was split (20 µL each) for data acquisition between platforms (see Materials and Methods) as similarly designed in a previous study^13^. Since qPCR is a sample interdependent technology where the relative quantity and normalized gene expression data rely on ΔCq values, the analysis from a single plate assures the best quality results by eliminating any bias from inter-plate variability^16, 17^. Therefore, all reactions for the study were pipetted into a single 96-well plate each for ddPCR and qPCR technologies.

### Assessment of primer efficiency, linear dynamic range and precision of ddPCR and qPCR technologies for low target concentration

**Figure 1:**
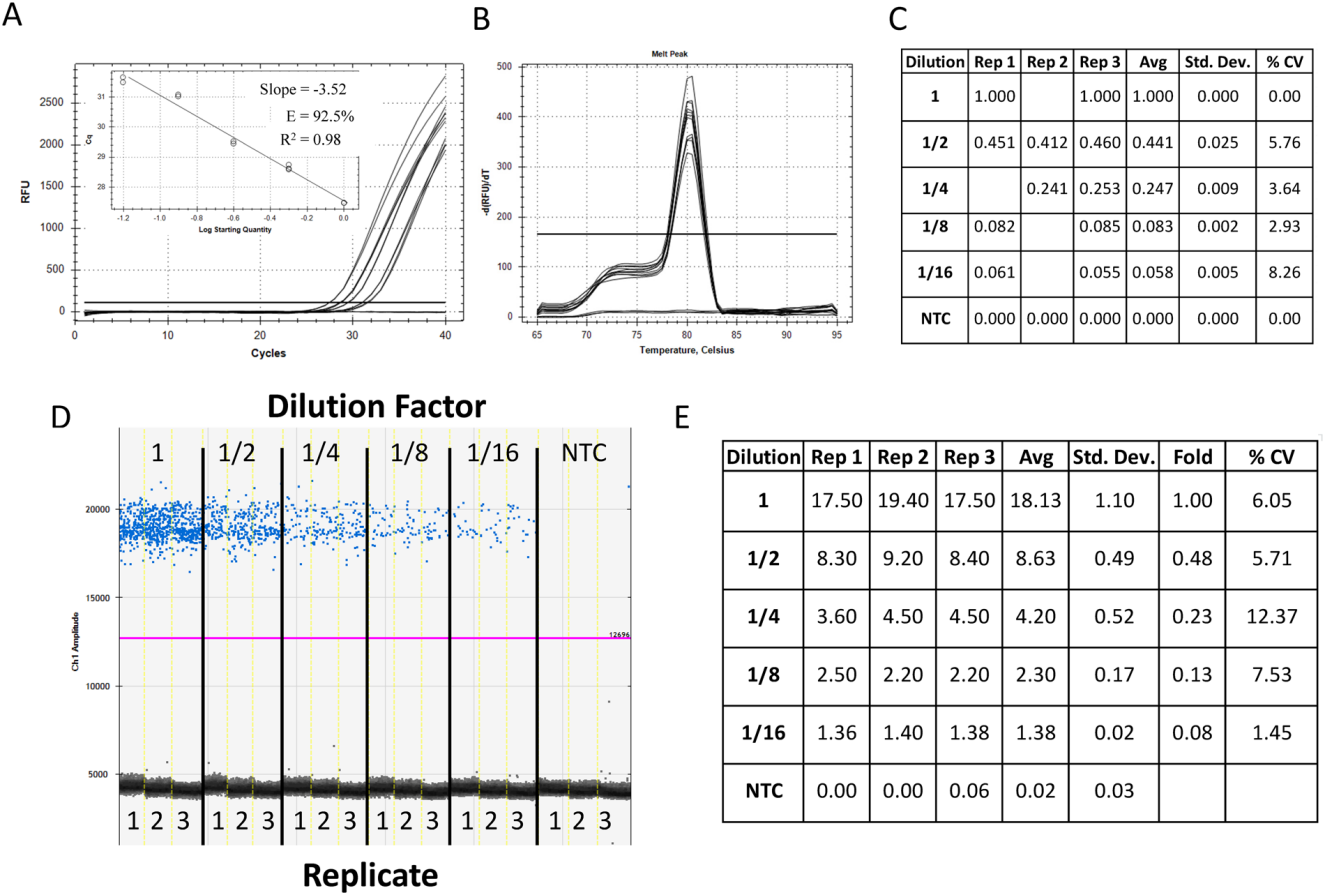
Assessment of primer efficiency, linear dynamic range and precision of ddPCR and qPCR platforms for low target concentration. Five reactions of 45 µL were prepared in triplicate from 1/2 serial dilutions of synthetic DNA in nuclease-free water with primers and ddPCR EvaGreen supermix. Each reaction mix was split for quantification in qPCR and ddPCR (20 µL for each platform). Amplification traces (**A**), standard curve (A inset), melt analysis (**B**) and tabulated relative fold difference (ΔCq) results (**C**) for qPCR. The ddPCR amplitude plot (**D**) and tabulated absolute concentration data (**E**). NTC: No Template Control; Dilution: Dilution factor of DNA samples; Rep: Replicate number; Avg: Average of the replicates; Std. Dev.: Standard Deviation between the replicates; % CV: % Coefficient of Variance (Std. Dev./Avg*100).

For qPCR, the primers gave good reaction efficiencies (between 90% and 110%) for low concentration samples diluted in water between 27 and 32 cycles (Fig. 1A inset) with a single melt curve peak (Fig. 1B) and a relative fold decrease of approximately 2-fold between each 1/2 dilution (Fig. 1C -“Avg”) with low variability between replicates (<15% CV). The ddPCR data generated from the identical reaction mixtures revealed good separation between the negative and positive droplets with few interface droplets (Fig. 1D) supporting good primer specificity and reaction efficiency. The absolute concentrations of DNA from ddPCR technology correlated with the 2-fold dilution factor used between the samples (Fig. 1E -“Fold”).

### Effect of consistent sample contamination between identical DNA dilutions for ddPCR and qPCR technologies

**Figure 2:**
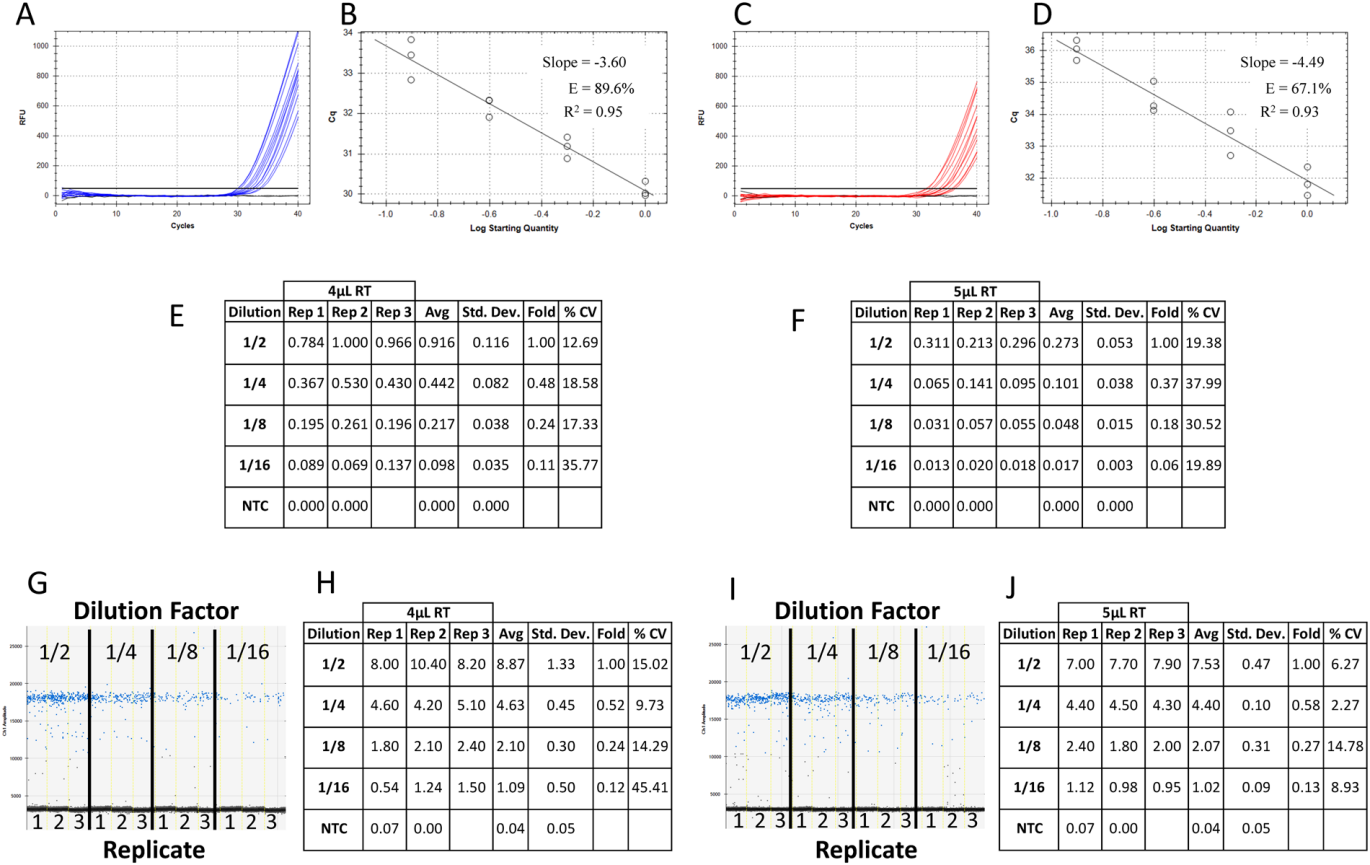
Effect of consistent sample contamination between identical DNA dilutions for ddPCR and qPCR technologies. Four reactions of 50 µL were prepared in triplicate from 1/2 serial dilutions of synthetic DNA in a background of either 10 µL (**A**) or 12.5 µL (**B**) of 1X reverse transcription (RT) mix with primers and ddPCR EvaGreen supermix. Each reaction mix was split for quantification in qPCR and ddPCR (20 µL for each platform containing either 4 µL or 5 µL of contaminating RT mix) and run on a single plate for each platform. Amplification traces (**A** and **C**), standard curves (**B** and **D**) and tabulated relative fold difference (ΔCq) results (**E** and **F**) were generated for qPCR. The ddPCR amplitude plots (**G** and **I**) and tabulated absolute concentration data (**H** and **J**) from the same reactions supplemented with 4 uL (**A,B,E,G,H**) and 5 uL (**C,D,F,I,J**) of RT mix were produced. NTC: No Template Control; Dilution: Dilution factor of DNA samples; Rep: Replicate number; Avg: Average of the replicates; Std. Dev.: Standard Deviation between the replicates; % CV: % Coefficient of Variance (Std. Dev./Avg*100).

Since the most common contaminant in a qPCR reaction is the reverse transcription (RT) mix from which the cDNA is synthesized, reaction mixtures containing a 1/2 dilution series of low concentration synthetic DNA supplemented with either 4 µL or 5 µL of RT mix were split between the qPCR (Fig. 2A–F) and ddPCR (Fig. 2G–J) platforms. For qPCR, the reaction efficiency was approximately 89.6% and 67.1% with 4 µL (Fig. 2B) and 5 µL (Fig. 2D) of RT mix respectively resulting in an approximate 2 Cq shift in the amplification curves (compare Fig. 2A – 30 to 34 Cq with 2 C – 31 to 36 Cq). This correlated to a perceived four-fold reduction in average relative quantity at each DNA dilution with increased RT mix (compare “Avg” between Fig. 2E and F at each dilution).

For ddPCR technology, the number of interface droplets between the positive (blue) and negative (black) amplitudes increased with the amount of RT contamination (compare Fig. 1D (none), Fig. 2G (4 µL) and Fig. 2I (5 µL)). However, the average absolute concentration was very similar for each respective dilution (compare “Avg” for the same dilution between Figs 1E, 2H and 2J).

### Effect of inconsistent sample contamination for the combined RT-contaminated samples at each DNA dilution for ddPCR and qPCR technologies without normalization

**Figure 3:**
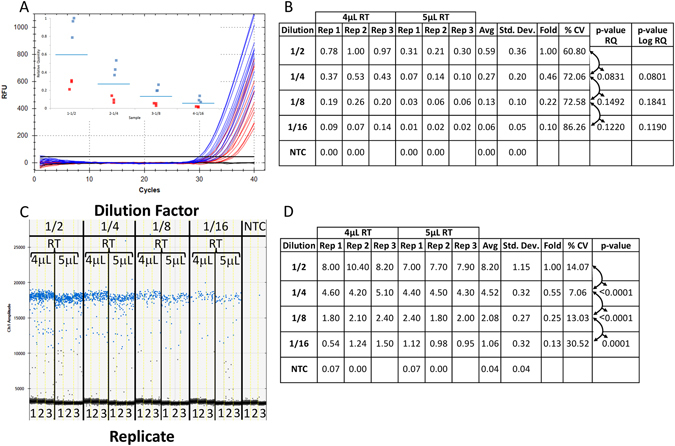
Effect of inconsistent sample contamination for the combined RT-contaminated samples at each DNA dilution for ddPCR and qPCR platforms without normalization. The reproducibility of the data between the combined reactions containing differing amounts of (RT) mix (Fig. 2) was examined for qPCR and ddPCR technologies. Amplification traces (**A**) and box plot (A inset) from the combined qPCR reactions supplemented with 4 uL and 5 uL of RT mix (blue and red traces and graphed points respectively) and tabulated relative fold difference (ΔCq) was produced (**B**). The amplitude plot and tabulated absolute concentration data from the combined ddPCR reactions supplemented with 4 uL and 5 uL of RT mix (**C**) and tabulated absolute concentration data (**D**) was generated. NTC: No Template Control; Dilution: Dilution factor of DNA samples; Rep: Replicate number; Avg: Average of the replicates; Std. Dev.: Standard Deviation between the replicates; % CV: % Coefficient of Variance (Std. Dev./Avg*100); p-value: based on a Student t-test of the Avg between each dilution.

To assess the effect of inconsistent contamination on qPCR and ddPCR, the data from the 4 µL and 5 µL RT-contaminated samples (Fig. 2) were combined at each dilution of synthetic DNA without normalization (Fig. 3). Since all samples for both levels of contamination were quantified from the same plate, the relative quantification method was used to assess the results. The combined results for each dilution of DNA and RT mix were assessed (Fig. 3A (4 µL - blue and 5 µL - red) and Fig. 3B (compare Rep 1 to 3 at each level of RT mix and “%CV” for the combined results). The separation between negative and positive droplets and associated quantitative data for the identical samples were also gathered using ddPCR technology (Fig. 3C and D).

### Inconsistent contamination levels on ddPCR and qPCR data with reference gene normalization

**Figure 4:**
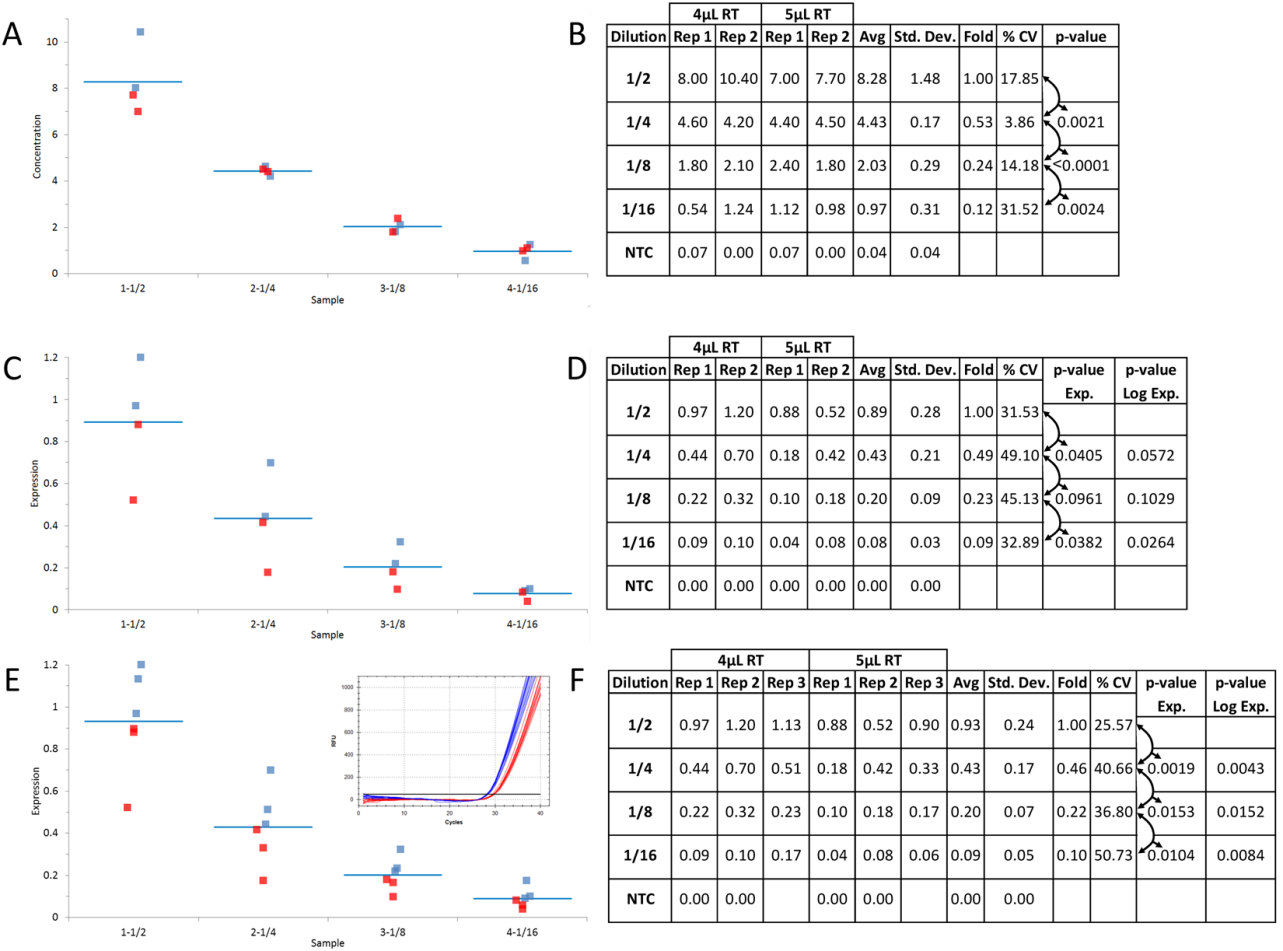
Reference gene normalization can improve qPCR precision depending on contaminant levels and number of replicate samples. The reproducibility of the data between the combined reactions ﻿at each 1/2 dilution of DNA﻿ (Fig. 2) was compared between ddPCR and qPCR technologies with normalization to the same synthetic target amplified from a consistent amount of DNA at each level of RT contaminant. Box plot (**A**) from four combined ddPCR reactions supplemented with 4 uL and 5 uL of RT mix and tabulated absolute concentration data from the combined ddPCR reactions (**B**). Box plot (**C**) from qPCR data generated from the same four reactions as described in A with normalization to the same target at a consistent DNA concentration between the RT contaminated samples (E inset) and tabulated normalized relative fold difference (ΔΔCq) (**D**). Box plot (**E**) from qPCR data generated from all six reactions as described in Fig. 2 with normalization to the same target at a consistent DNA concentration between the RT contaminated samples (E inset) and tabulated normalized relative fold difference (ΔΔCq) (**F**). Blue and Red traces and graphed data points from qPCR and ddPCR reactions supplemented with 4 uL and 5 uL of RT mix respectively. NTC: No Template Control; Dilution: Dilution factor of DNA samples; Rep: Replicate number; Avg: Average of the replicates; Std. Dev.: Standard Deviation between the replicates; % CV: % Coefficient of Variance (Std. Dev./Avg*100); p-value: based on a Student t-test of the Avg between each dilution.

A consistent amount of synthetic DNA was introduced to reactions under the identical conditions and contaminant levels used for the diluted samples to mimic a reference gene (Fig. 4). The data from four of the replicates (ie: two from each of the 4 µL and 5 µL of RT mix contaminated samples) were combined at each dilution of synthetic DNA for both ddPCR technology without normalization and qPCR with normalization (Fig. 4A and C respectively -4 µL (blue) and 5 µL (red) and Fig. 4B and D). The full cohort of six samples was also quantified using qPCR with normalization (Fig. 4E and F).

The reference gene data for qPCR shifted from an average of 28.5 to 30.0 Cq values between the two levels of RT mix contamination corresponding to an approximate 2.8 fold (ie: 2^(30–28.5)^) or 280% contamination effect on the data (Fig. 4E -inset -4 µL (blue) and 5 µL (red)). Conversely, the ddPCR results showed very little variability between the two levels of contamination for the same samples with average concentrations of 34.0 and 32.0 copies per µL (data not shown) at the two levels of contaminant giving a 5.9% (ie: (1-32/34)*100) difference and hence no significant effect of contamination.

## Discussion

### qPCR and ddPCR technologies give comparable results for identical samples with low levels of contamination

The data generated from standard curves of 1/2 serially diluted DNA in water gave the expected results with excellent precision, reaction efficiency and similar dynamic range for both ddPCR and qPCR platforms (Fig. 1). Thus, for samples that contain very low or no levels of background contaminants, ddPCR and qPCR technologies give similar performance for absolute and relative quantification. However, in the case of miRNA and mRNA, the components of the reverse transcription (RT) reaction can also act as inhibitors of Taq polymerase, altering reaction efficiency and the associated Cq values to give artifactual qPCR data if not adequately diluted (Fig. 2)^18^.

### Consistent sample contamination within a set of conditions gives comparable data quality between qPCR and ddPCR platforms

Since qPCR data are calculated from Cq values that are measured from individual amplification curves, any changes in the chemical and/or protein contaminants altering Taq polymerase enzymatic activity and primer binding will change the Cq values between the samples independent of the absolute amount of DNA^18–20^. The most susceptible samples include those with high levels of impurities and low target concentrations because these samples cannot be adequately diluted to eliminate the effect of contamination on data quality^1, 21^.

The effect of the contamination was evidenced by the poor separation between the amplification curves and reaction efficiency well below 90% for the 5 µL of RT contaminant (Fig. 2C and D) resulting in an approximate 2 Cq shift at each dilution between the two levels of RT mix (compare Fig. 2A and B with 2C and D). Although the 1/2 dilution series from 4 µL of RT contaminant produced the predicted 2-fold reduction in average relative quantity with good precision (Fig. 2E - “Fold” and “% CV”) there was generally higher variability and less precision at the 5 µL level of contaminant particularly at the 1/16 dilution which decreased by 3-fold from the 1/8 dilution. In contrast, the ddPCR platform gave a consistent absolute concentration for each dilution of DNA between the two levels of RT mix contaminant with good precision and the expected 2-fold difference (compare Fig. 2H and J-“Avg”, “% CV” and “Fold”).

Thus, presuming a given set of samples contains the same level of background contamination, qPCR and ddPCR technologies produce similar results for gene expression even when contaminant levels are high enough to affect reaction efficiency. However, there are frequently subtle differences in the level of impurities between samples even under highly rigorous RNA extraction procedures that can alter Taq activity, imparting gross variability on the resulting data^18, 19^.

### Variable contamination between samples gives high variability and inconsistent data for qPCR whereas ddPCR results remain consistent and reproducible

Combining the data from Fig. 2 gave six replicate samples for each dilution containing the identical amount of DNA but variable levels of RT mix contaminant (Fig. 3). For qPCR, the amplification curves and resulting Cq values were highly variable within each dilution between the 4 µL and 5 µL of RT contaminant (Fig. 3A - 4 µL (blue) and 5 µL (red)). Thus, the relative quantity between each replicate differed by approximately 4-fold giving two distinct clusters of triplicate data points at each dilution (Fig. 3A inset -(4 µL (blue) and 5 µL (red)) and Fig. 3B - compare each of the three replicates “Rep” for each dilution between the RT contaminant levels). Although the average relative quantity (Fig. 3B - “Avg”) gave a consistent 2-fold difference between each 1/2 dilution (“Fold”), the variability was very high ranging from 60% to 87% (“% CV”) with no statistically significant differences between each dilution for qPCR (“p-value” ranging from 0.0831 to 0.1492 and log transformed from 0.0801 to 0.1841).

For ddPCR technology, the combined results at the two levels of contaminant gave consistent separation between negative and positive droplets and minimal interface droplets at each DNA dilution (Fig. 3C). The absolute concentrations were highly reproducible between the replicates for each DNA and contaminant level (Fig. 3C - “Rep”) with an average concentration (“Avg”) that was precisely decreasing by 2-fold (“Fold”) as expected. More importantly, the variance between the replicates was much lower than with qPCR (between 7% and 30% for ddPCR technology versus 60% to 87% for qPCR (compare Fig. 3B and D - “% CV”) with strong statistically significant differences (Fig. 3D - p-value < 0.0002) between each dilution.

### Reference gene normalization can improve qPCR precision depending on contaminant levels and number of replicate samples

In order to mimic the perfect reference gene, the same synthetic DNA sample used for the 1/2 dilutions throughout this study was introduced at the same concentration into reaction mixtures containing the two different levels of RT mix on the same plate as the 1/2 dilutions. Since many labs performing cell-based assays use smaller sample sets (<5 biological replicates), a subset of four samples per DNA dilution consisting of the first two replicates (ie: Fig. 3B and D - Rep 1 and 2) from each of the 4 µL and 5 µL levels of RT-mix contaminant were analyzed (Fig. 4A–D). The ddPCR absolute concentration data remained accurate and precise without normalization as shown by the tight clustering of the four data points around each dilution (Fig. 4A - 4 µL (blue) and 5 µL (red)) with a precise 1/2 fold decrease, low % CV and high statistically significant differences (p-value ≤ 0.0024) (Fig. 4B). However, the normalized relative qPCR results from the identical four samples gave variable clustering at each dilution (Fig. 4C -4 µL (blue) and 5 µL (red)) with no statistically significant differences between 1/4 and 1/8 DNA dilutions (p-value of 0.0961) and between the 1/2 and 1/4 and 1/8 dilutions with log transformed data (p-value of 0.0572 and 0.1029 respectively) (Fig. 4D).

When the full set of three replicates from each DNA dilution and RT-mix background (ie: six samples total per dilution) were tabulated, the normalized relative expression values between the different levels of RT-mix were much tighter (varying by about 1.5-fold between the two levels of contaminant) than those from the non-normalized data which varied by about 4-fold (compare Fig. 3A inset with 4E (4 µL (blue) and 5 µL (red)) and Fig. 4F with 3B - “Rep” within each dilution). Furthermore, the normalized data for qPCR gave ﻿﻿﻿more precise and﻿ statistically significant data for the average of the six replicates between each dilution for the identical samples (compare Fig. 3B with 4F - “% CV” and “p-value”). This underlines the effect of reference gene normalization where the 2.8 fold variance in reference gene expression between the two levels of contaminant (Fig. 4E - inset - 4 µL (blue) and 5 µL (red)) effectively normalized the 4-fold (non-normalized data – Fig. 3A and B) to a much tighter 1.5 fold (Fig. 4E and F).

Although the p-values obtained for qPCR with normalization were below 0.05 for the six samples (Fig. 4F), the preference by many labs is to achieve higher levels of reproducibility such that the reported p-values are at or below 0.001^22^. The qPCR results could not meet this more stringent criterion even with normalization for the small sample set (Fig. 4D – all p-values > 0.01) and for the large sample set (Fig. 4F – most p-values > 0.008). Whereas the ddPCR data for both small and large sample sets gave p-values either well below or close to 0.001 for all dilutions without normalization (Figs 3D and 4B).

Reference gene normalization was not required in this experiment for ddPCR technology because there was virtually no effect of contamination and a measured amount of DNA was applied to each sample (see results section) but this does not mean that reference genes are never required for this application. The protein and chemical contaminants that vary between RNA extracts from biological samples can not only partially inhibit the qPCR reaction but also the reverse transcriptase leading to variable levels of input cDNA in the ddPCR reaction^23^. Hence, reference gene normalization is always recommended for gene expression analysis with ddPCR technology.

### Criteria for selecting between qPCR and ddPCR platforms for absolute quantification, gene expression or miRNA analysis

Given the large diversity of samples used in life sciences, some general criteria can be applied to selecting the appropriate technology for gene expression analysis as follows:

1. Always begin a study by testing each primer pair in a 1/10 diluted, pooled cDNA or gDNA sample (using individual samples from each treatment group in the study set) in duplicate with qPCR under optimized thermocycling conditions and a no template control for each target.
2. Presuming good, sigmoidal amplification curves are obtained with single melt curve peaks for each primer pair, run the duplicate samples from the qPCR reactions on a gel to assess the molecular weight and identity of the amplicon and sequence if necessary.
3. All targets must then be assessed for reaction efficiency with a standard curve using the same pooled gDNA or cDNA sample from point 1 serially diluted based on the Cq value obtained from point 2 starting from the concentrated sample plus seven dilutions^7^.
4. Any target that passes point 2 but fails point 3 should be ported to ddPCR technology for optimization^13^. Individual samples for any target that passes points 2 and 3 should be diluted appropriately according to the associated standard curves^7^ and quantified using qPCR but if the resulting data give high variability and poor p-values, these targets may also be good candidates for ddPCR technology but should also first be optimized^13^.
5. Primers that fail optimization using ddPCR technology should either be redesigned or the experimental design and samples should be re-assessed and undergo troubleshooting^7^.

qPCR is an excellent tool for the detection of 2 fold or greater expression differences with good statistical significance above about 100 copies (<30 Cqs) in applications such as gene expression and/or﻿ miRNA analysis and absolute DNA quantitation. Although reference gene normalization serves the important purpose of correcting for differential loading of cDNA between samples, it may not reduce contaminant dependent data variability observed with qPCR (Figs 3 and 4) which can only be addressed through sample dilution^7^. Thus quantification of low expressing/abundant targets using qPCR is challenging because sample dilution is not possible to adequately minimize the effect of contaminants while maintaining quantifiable levels of cDNA. ddPCR technology permits quantification of these samples with excellent precision.

## Methods

### Primers, template and Reverse Transcription Mix

Primers and template were sourced from the EvaGreen ddPCR™ Demonstration Kit (Bio-Rad: 186–4029). Primers were diluted according to the kit instructions and template was diluted by approximately 400 fold to achieve the starting concentration used in each experiment. The iScript™ Reverse Transcription (RT) Supermix, 100 × 20 µl rxns (Bio-Rad: 170–8841) was diluted to 1X with corresponding volumes transferred to each reaction (see Results and Figures).

### ddPCR and qPCR reactions

50 µL reaction mixtures containing RT mix, primers, template and QX200™ ddPCR™ EvaGreen Supermix (Bio-Rad: 186–4034) were divided 20 µL each between ddPCR (QX200 Droplet Digital PCR (ddPCR™) System – Bio-Rad) and qPCR (The CFX96™ Touch System – Bio-Rad) platforms for quantification. For ddPCR technology, droplet generation and transfer of emulsified samples to PCR plates was performed according to manufacturer’s instructions (Instruction Manual, QX200™ Droplet Generator – Bio-Rad).

The cycling protocol was identical for both platforms with a 95 °C enzyme activation step for 5 minutes followed by 40 cycles of a two-step cycling protocol (95 °C for 30 seconds and 60 °C for 1 minute). For ddPCR technology, the ramp rate between these steps was slowed to 2 °C/second while for qPCR, the maximum ramp rate was employed. Post cycling, a standard melt curve protocol was applied for qPCR (a single step of 94 °C for 10 seconds followed by a melt curve from 65 °C to 95 °C with a plate read at 0.5 °C increments after a dwell time of 5 seconds at each temperature). For ddPCR technology the post-cycling protocol was in accordance with the kit instructions (Bio-Rad – 186–4034).

### ddPCR and qPCR plate set up and data processing

Each reaction mixture (see previous section) was split between one plate each for ddPCR (ddPCR™ 96-Well Plates: Bio-Rad - 12001925) and qPCR (Hard-Shell® 96-Well PCR Plate: Bio-Rad - HSP9601). The qPCR plate was sealed (Microseal® ‘B’ PCR Plate Sealing Film: Bio-Rad - MSB1001) and run in qPCR. The ddPCR plate was sealed with a foil heat seal (Bio-Rad - 181–4040) and the PX1™ PCR Plate Sealer (Bio-Rad - 181–4000).

For qPCR, the relative quantity and normalized expression data were processed using CFX Manager (v.3.1). For ddPCR technology, the absolute quantity of DNA per sample (copies/µL) was processed using QuantaSoft (v.1.7.4). For both ddPCR and qPCR, the data were exported to Microsoft EXCEL for further statistical analysis using the Analyze IT plugin (Analyse-it for Microsoft Excel (v.2.20) Analyse-it Software, Ltd. http://www.analyse-it.com/; 2009).

### Data and Statistical Analysis

All statistically analyzed data conformed to a normal distribution as determined using a Shapiro-Wilk test. A Student’s t test was then used to assess the statistical significance between the two-fold dilutions of DNA for each experiment. The %CV (Standard Deviation/Mean *100) was also used to assess variability. Finally, since it is generally accepted that qPCR derived gene expression data are log transformed for statistical analysis^24^, the log transformed normalized gene expression and relative quantity data from each biological replicate were also normality tested (Shapiro-Wilk) and then Student’s t tested for statistical significance.
